# Liquid crystallinity of carbon nanotubes

**DOI:** 10.1039/c8ra00879e

**Published:** 2018-04-26

**Authors:** Chunrui Chang, Ying Zhao, Ying Liu, Libao An

**Affiliations:** North China University of Science and Technology, College of Science Tangshan 063009 China changchunrui@ncst.edu.cn +86 18032513036; Hebei Milestone Electronic Material Limited Company, Research and Development Department of Liquid Crystal Mixture Shijiazhuang 050600 China; North China University of Science and Technology, College of Mechanical Engineering Tangshan 063009 China

## Abstract

In this review, we first briefly recapitulate the orientation characteristics of liquid crystalline carbon nanotubes (CNTs), emphasizing their inherent properties. Both the high Young's modulus and the strong attractive interaction between them make the liquid crystallinity apt to show splay deformations (splay defects). It is these defects that often produce apparent low-order structures for long and deformed nanotubes. However, the application of doping, shearing, magnetic or electric fields will be efficient routes toward highly ordered CNT assemblies from such defects. Then, we describe the electrical behavior of CNTs in the electric field, which combines desirable features of the CNTS with those of classical liquid crystals (LCs). An electric field will generate an induced dipole moment on CNTs and align them in the field direction, minimizing the dipolar energy. Finally, we review the potential application of CNTs in the area of liquid crystal displays (LCD). In the LC cell unit, CNTs as dopants in LC layers can have compatible stability with LCs, with the orientation consistent and with surprising complementary advantages. And also CNT films as nanostructured electrodes can substitute ITO electrodes in the LC cell unit, exhibiting a strong electrical anisotropy due to their excellent axial conductivity. Furthermore, CNT films as an alignment layer have the potential to replace the traditional PI film, aligning LC molecules effectively along the direction of the nanotubes. Besides, CNTs acting as polarizers can absorb or transmit incident light when the electric vector propagates parallel or perpendicular to the nanotube axis. All of these applications demonstrate that CNTs in LC ordering will effectively improve the performance of materials and their related devices. Thus, we should improve the ordering of CNT assemblies as far as possible, which is critical to make full use of their exceptional axial properties and further to develop novel materials and applications successfully.

## Abstract 2

In this review, we first briefly recapitulate the orientation characteristics of liquid crystalline carbon nanotubes (CNTs), emphasizing their inherent properties.

## Graphical abstract

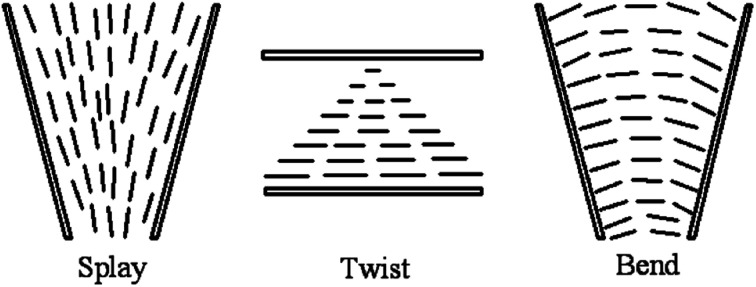

## 1. Introduction

Since their discovery, the unique anisotropic structure of CNTs with a large aspect ratio displays extreme anisotropic electrical, magnetic, and optical properties, and they are expected to be very good electrical, magnetic, and optical conductors along the axis direction.^1,2^ For this reason, they are promising anisotropic nanomaterials for a variety of applications such as strong and lightweight composites, sensors, electronic devices, conductive inks, substrates for tissue engineering, *etc.*^1–3^ And the anisotropic nature can be best preserved and explicitly conceived when all the CNTs are aligned in the same direction in larger structures of them. As a result, it is always necessary to consider the orientation of CNTs when they are considered for both scientific studies and practical applications. Control over the tube orientation is thus highly desirable for the achievement of the unique axial characteristics and the corresponding applications. There are a handful of experimental technologies available to align a single CNT or an array of CNTs along a predetermined orientation. These techniques can be categorized into two groups: (a) *in situ* techniques where alignment is achieved during the CNT growth process and (b) *ex situ* techniques where alignment is achieved afterwards such as during the device integration process. The *in situ* techniques always come first by the consideration of a manufacturer for the benefit of straightforwardness and simplicity. And the *ex situ* techniques are free of growth restrictions such as substrate material and temperature. *Ex situ* assembly of CNTs can be effectively achieved by electric fields, magnetic fields, force fields, and others. In these methods, each method has its own limitations and difficulties. But even so, CNT alignment is critically required for diverse applications^4–6^ and achieving the desirable ordering of such anisotropic materials is a considerable challenge.

**Fig. 1:**
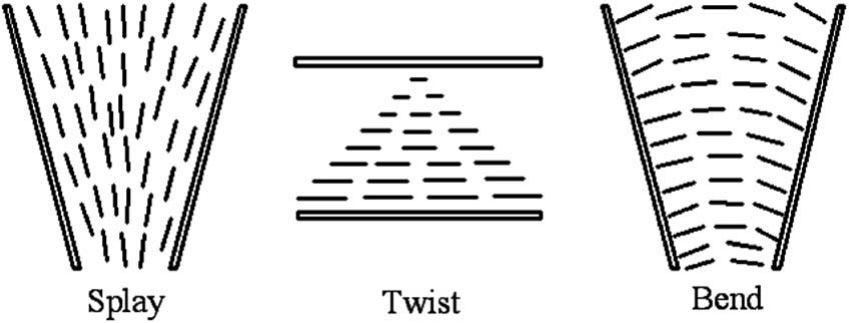
The three fundamental types of distortions in a nematic liquid crystal. Adapted from ref. 9. Copyright 1977, the Press Syndicate of the University of Cambridge.

CNTs are generally depicted as long cylindrical rods and reminiscent of liquid crystals (LCs) that are complex fluids with microstructural ordering. A substance in this state is strongly anisotropic in terms of mechanical, electric, optical, magnetic and other properties, and exhibits a certain degree of fluidity like that of an ordinary liquid. Maybe we can further understand the orientation of nanotubes and achieve the desirable ordering with the help of this similarity. Liquid crystalline states may be brought about by purely thermal processes (thermotropic LCs) or by the influence of solvents (lyotropic LCs),^7^ and CNT liquid crystals belong to the latter. Based upon the basic physics of molecular LCs, liquid crystallinity of CNTs and the related ordering can be further acquired. As we know, the nature of molecular alignment in LC media is discussed in terms of the director **n**, which refers to the direction of the long axes of more or less parallel molecules in a small local volume of the material. The macroscopic condition of the LC is thus characterized by the vector field of this director, *i.e.*, **n**(**r**) at each spatial position. Under the condition of continuous elastic theory, the physical distortions of a LC of the nematic (the most common LC phase) category can be described by the excess free energy that in general is composed of only three independent terms:^8^1
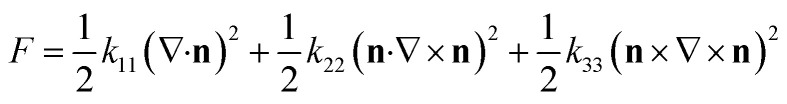
where *F* is the Frank distortion free energy per unit volume and *k*_11_, *k*_22_, and *k*_33_ are Frank elastic constants of modes of splay, twist, and bend, respectively. A representation of the three fundamental distortions is given in Fig. 1.^9^ The moduli *k*_*ii*_ are extremely weak and the low values of them are responsible for the unusual sensitivity of LCs to external stimuli and boundary conditions. It should be noted that the three moduli considerably simplify the description of material anisotropy and response. Then when considering a planar sample in which the director is confined in the plane, the observation in the microscope will involve only splay and bend distortions, as well as only the splay–bend elastic anisotropy will need to be taken into account. Such known nematic LC often has a high degree of long-range orientational order of the molecules and such property of orientation is often called LC ordering (aligning effect).

Here we describe the liquid crystalline behavior of CNTs, combining desirable features of CNT suspensions with those of classical LCs. Due to the common anisotropic shape CNTs (high aspect ratio, *L*/*D*) will usually form the lyotropic nematic LC phase when they are suspended in a liquid medium at sufficiently high concentration.^10–22^ And the lyotropic CNT liquid crystals can also take place under such specific conditions as the action of external fields: magnetic,^23^ electric,^24^ shear,^25^ spinning,^26^ or drawing.^27^ The formation of LC ordering for CNTs will facilitate an opportunity to develop novel materials and applications. However, CNT liquid crystals often exhibit a number of topological defects too due to the length and waviness of them.^28–30^ Although such defects are unfavorable to realize the full performance of CNTs in most cases, they can be described by Frank elastic constants *k*_*ii*_ that can give how individual nanotubes are arranged in the defects similar to traditional LCs. The exploration of relationship between Frank elasticity to nanotubes and the defective properties will contribute to their liquid crystalline formation and the related applications, especially for the combination with LCs in displays.^31–45^ In the area of liquid crystal display (LCD), combination of CNTs and LCs is expected to demonstrate exceptional novel or surprising properties. On one hand, LC molecular liquid can facilitate large-scale uniform alignment of CNTs along their director even for relatively high concentration.^46–52^ And conversely the LC ordering can be further improved by the addition of nanotubes, *i.e.* CNTs may well modify the LC ordering.^53^ It is the role of the aromatic systems in LC-CNT mixtures that play in the behavior of dispersing and aligning.^53,54^ On the other hand, CNTs, viewed as highly anisometric rigid rod-like particles, can be manipulated by electric or magnetic fields due to the dielectric and magnetic anisotropy, which is the basis for their use in modern display devices.^55–58^

For all of these reasons, the interest in liquid crystallinity of CNTs is quite active. This paper is a review of the literature that deals with liquid crystallinity of CNTs and the relevant technological applications. In the following chapters, we will focus on such fundamental facets as liquid-crystal theory of CNTs, liquid-crystalline defects of CNTs and the liquid-crystalline applications in the area of display. Finally, we give a summary and outlook.

## 2. Liquid-crystal theory of CNTs

We have known that CNTs will form a lyotropic liquid crystalline phase when they are suspended in a solvent at a sufficiently high concentration. Generally, the liquid crystalline origin of CNTs can be understood using the steric theory of rigid rod-like LCs,^59^ such as the tobacco mosaic virus (TMV)^60^ and some rigid polymers.^61–64^ Based upon the simple steric model, the molecular configurations are dominated by steric effects, depending on the concentration. In a dilute solution, the molecular configuration behaves isotropic because of the large free volume and in a concentrate solution, the system becomes liquid crystalline because of the inhibited free volume. Here the nanotubes exhibit the long-range orientational order with the thermodynamic stability, *i.e.* the loss of orientational entropy from the minimization of the excluded volume has been compensated by the associated increases of packing entropy and then thermodynamic entropy of the system reach the maximum.

**Fig. 2:**
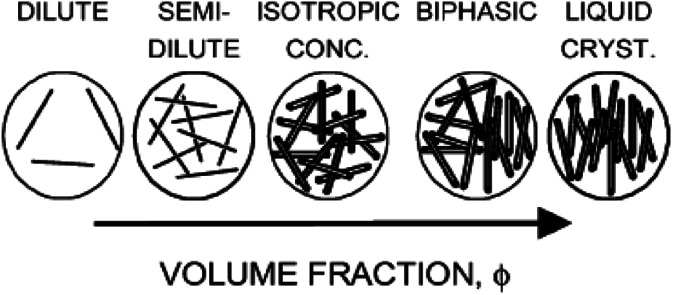
Phase behavior of solutions of rigid rods. Adapted from ref. 20. Copyright 2004, Macromolecules.

As shown in Fig. 2,^20^ with increasing concentration, the system will undergo the dilute solution where individual rods do not interact with each other, the semi-dilute solution, the isotropic concentrated solution where the isotropic configuration is favored, the biphase where the isotropic phase is equilibrium with the liquid crystalline phase, and the fully liquid crystalline phase where the parallel configuration is favored. In addition, the prerequisite for CNTs to form lyotropic liquid crystalline phase (primarily of nematic type) with good quality is to maintain a sufficiently good dispersion. CNTs are notoriously difficult to disperse in any medium and the long-term stability of the dispersion is extremely difficult to achieve due to the strong attraction from physical adsorption of van der Waals forces between tubes.

**Fig. 3:**
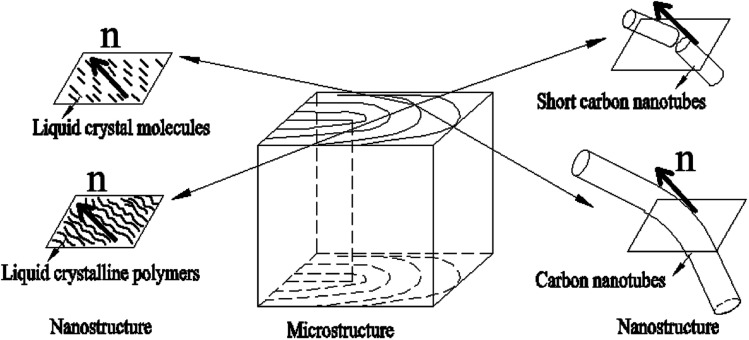
Outline of the different scales of nematic CNTs, conventional LCs, and liquid-crystalline polymers. Adapted from ref. 65. Copyright 2008, Advanced Materials.

And further since the anisotropic building blocks of CNTs are usually analogous to rod-like liquid crystals, short CNTs will behave like rigid rods and the longer ones are more like flexible polymers or inorganic nanowire. As shown in Fig. 3 (a planar-defect structural model),^65^ CNTs are frequently likened to traditional LCs or liquid crystalline polymers. In the liquid crystalline microstructures, only an individual CNT will be corresponding to the nanostructure of molecule LCs due to the large size of them. Short CNTs have the similar director **n** to conventional LCs and for long ones, the director **n** will become a tangent on an individual bending nanotube. The liquid crystalline alignment of CNTs is thus generally at the levels from a few to hundreds of micrometers.

Bending nanotubes have the energy barrier for their liquid crystallization, which does not need to address in molecule liquid crystalline systems. As a consequence, both of the optical texture and SEM images of such liquid crystalline microstructures about defects have significantly different properties from conventional LCs. In the following section, we will provide systematic discussions about these microstructural defects to further understand details of their liquid crystalline behavior.

## 3. Liquid-crystalline defects of CNTs

**Fig. 4:**
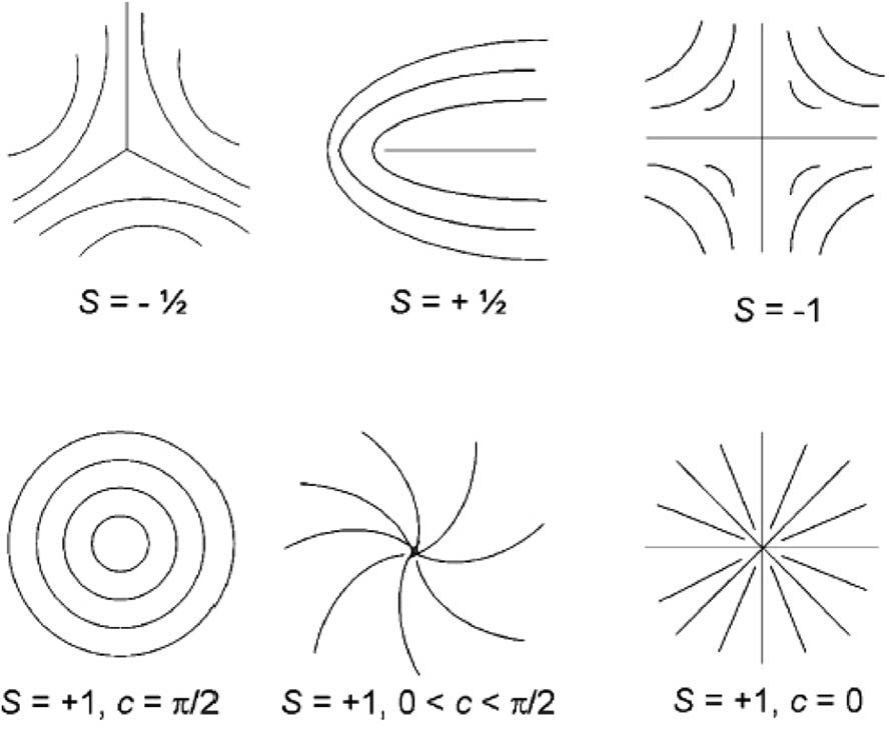
Molecule trajectories in a 2D ordered LC associated with defects of *s* = ±1/2 and ±1. Adapted from ref. 66. Copyright 2008, Small.

In the director field, conventional LC will almost always contain defects due to the high sensitivity to external forces, and these defects are often distinguished by the index *s* (called the strength of the defect). Some examples of the molecular orientation in the neighbourhood of a defect with a few values of *s* are shown in Fig. 4.^66,67^ Since the energy of a single defect is proportional to *s*^2^, only defects of strength *s* = ±1/2 and ±1 can be observed as the low-energy modes, producing relatively lower energy states. In fact, ±1/2 defects are more energetically favored and more often observed than that with *s* = ±1. In addition, as can be seen in the figure there may be different patterns for the defect of *s* = 1 due to the director orientation with different orientation angels *φ* and polar angels *θ* in an approximate relation of *φ*(*θ*) = *sθ* + *c* (*c* is the constant parameter). The inspection of defects can determine the fundamental symmetry of an underlying liquid crystalline material.

**Fig. 5:**
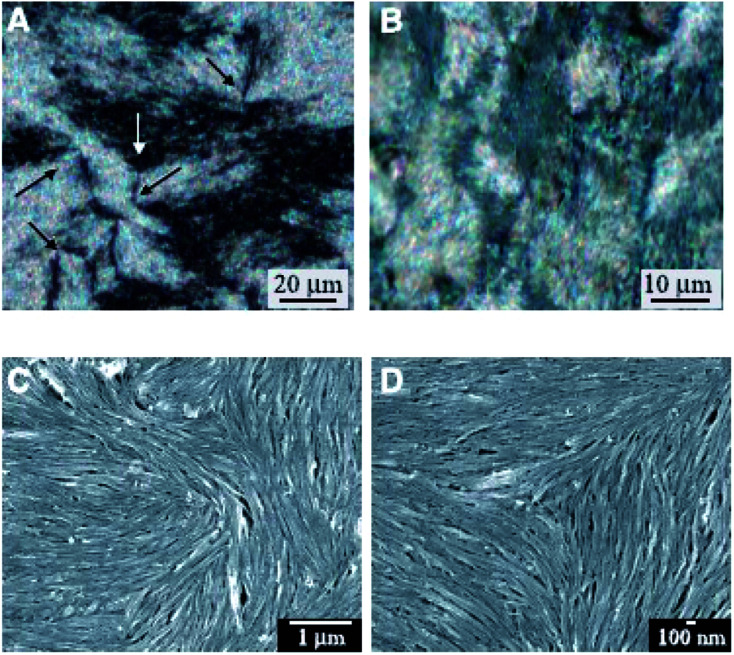
Optical images and SEM images of a nematic liquid crystalline phase. Adapted from ref. 68. Copyright 2003, Science.

Then in CNT liquid crystals, defects will also exist similar to conventional LCs by the nature of deformations or waviness of them. They often exhibit only small monodomains and contain a high density of defects corresponding to lines of singularity in the alignment. Here the relevant examinations will be further taken by means of polarized optical microscope and scanning electron microscope (SEM), as is shown in Fig. 5.^68^

From these characterization results we have successfully acquired the nematic liquid crystalline structure of nanotubes. First of all, optical images formed from CNT entities (Fig. 5(A) and (B)) have presented the characteristic Schlieren texture of a nematic. Then due to the instrumental resolution sufficient to view individual nanotubes SEM images (Fig. 5(C) and (D)) have further provided such microstructures for details. Both of the two types of characterization methods are very popular to determine the forming mechanism and physical properties of CNT liquid crystals. Based upon the continuous elastic theory described above, such liquid crystalline defects of nanotubes are expected to be closely related to Frank elastic constants *k*_*ii*_ that will critically dictate the behavior of a CNT nematic. Moreover, determination of *k*_*ii*_ by means of defects will further provide theoretical basis for the related application of CNT liquid crystals in many fields involving carbon nano-composites or carbon nano-array production or electronic devices. Therefore in the following two chapters, the two types of characterization results will be introduced for further details separately.

### 3.1 Optical textures of liquid-crystalline defects of CNTs

**Fig. 6:**
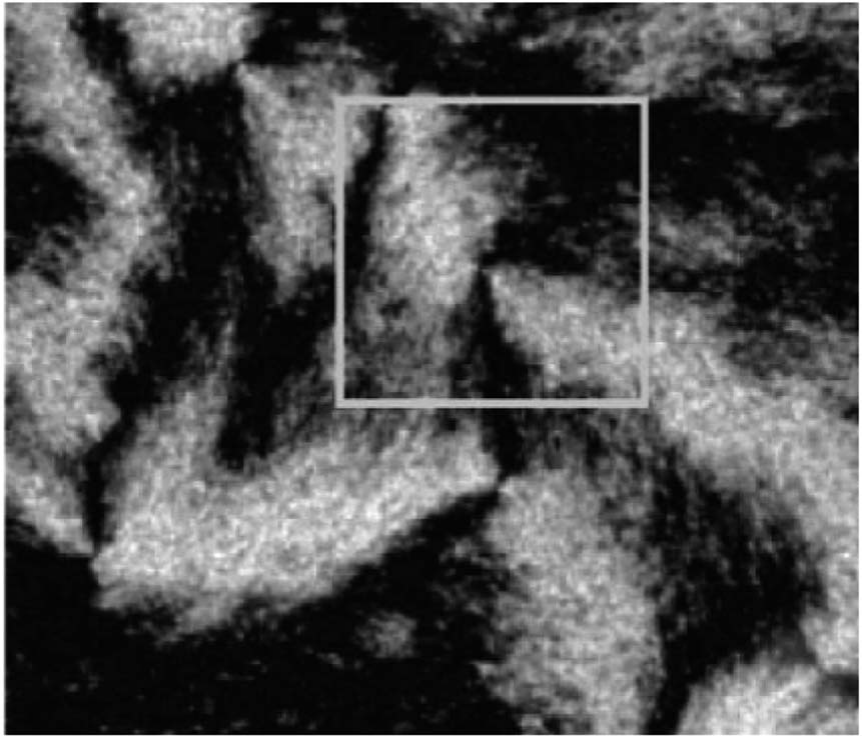
The typical Schlieren texture with crossed polarizers indicated by the two-armed brushes in the white box corresponding to defects. Adapted from ref. 69. Copyright 2005, Macromolecules.

A system of black stripes (the so-called ‘Schlieren texture’) is a most common optical texture of nematic LCs under crossed polarizers. As can be seen in Fig. 6,^69^ such Schlieren texture always contains distinctive “brushes” emanating from singularities and we will cover this at length.

The Schlieren texture has been obtained in reflected polarized light with the polars crossed (bireflection). It exhibits distinct black brushes that emanate from singular points (called ‘nodes’) and locate to the regions where the director (or the local optical axis) is either parallel or perpendicular to the plane of polarization of the incident light. Obviously, such singular nodes are connected by the black brushes, very characteristic for the nematic phase. In fact, some nodes have four black brushes while others have only two, and the strength *s* of a defect is defined as 1/4(number of brushes). The nodes with *s* = ±1 are often called defect points, whereas any node with *s* = ±1/2 must be the end point of a defect line of the same strength. And further when the crossed polars rotation happens the brushes will also rotate. For example, the brushes from defects of strength *s* = ±1/2 will rotate at twice the angular velocity of the crossed polars, as well as they will rotate in the same or opposite sense as the polars depending on the positive or negative value of the strength *s*. However, details of liquid crystalline defect structure of how CNTs are aligned need to be further examined using SEM at a resolution sufficient to view individual nanotubes.

### 3.2 SEM images of liquid-crystalline defects of CNTs

**Fig. 7:**
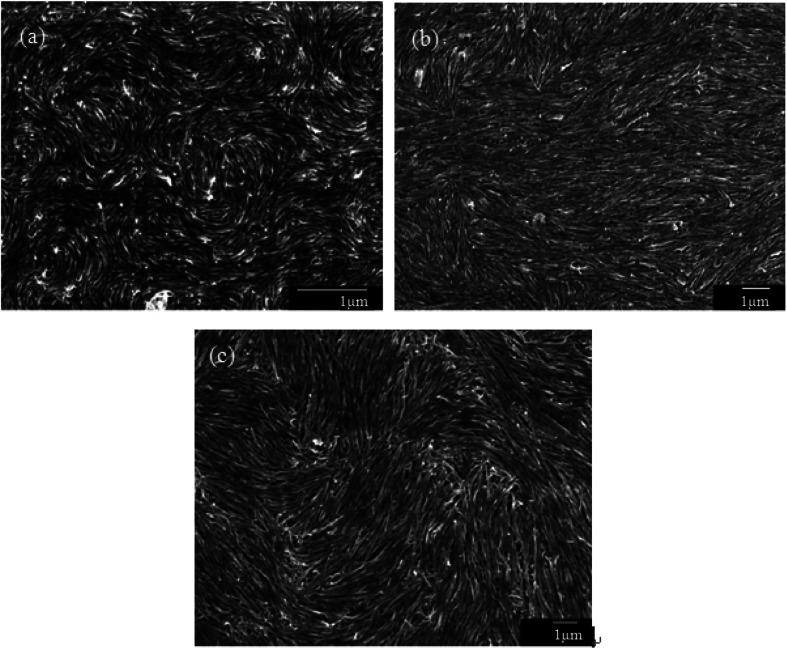
SEM images of a nematic liquid crystalline phase of (a) short and thin CNTs, (b) short and thick CNTs, and (c) long and thick CNTs with a group of defects showing the dominant ±1/2 defects. Adapted from ref. 65. Copyright 2008, Advanced Materials.

The observed liquid crystalline defect structure by SEM can describe how individual nanotubes contribute to distortions of the director field surrounding the defects. Here all of these SEM images have originated from the dried nematic films and correspond to the optical textures. Since the observed nanotubes are mainly constrained to lie in the plane of the film, only the splay and bend distortions (described by Frank elastic constants analogous to LCs, *k*_11_ for splay and *k*_33_ for bend) will be presented. And due to the high resolution of the electron microscope, such defects composed of nanotubes will clearly show how they cope with the bend and splay distortions. On one hand, only defects of strength *s* = ±1/2 and ±1 can be observed, and the defects with strength *s* = ±1/2 will be energetically more favored than ±1 defects (Fig. 7). On the other hand, the imaging of individual nanotubes in the defects about how to cope with the bend and splay distortions also depends on the positive or negative value of the strength *s*. For instance, the Frank bend distortion is energetically favored for +1/2 defects, whereas in −1/2 defects the splay packing will become more favorable.

**Fig. 8:**
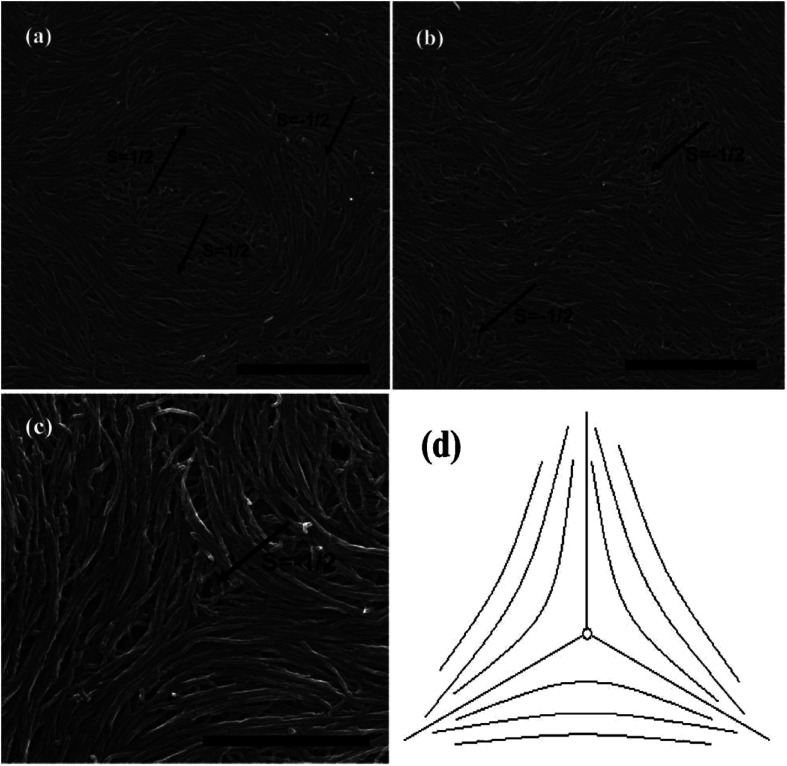
SEM images of the splaying defective cores formed by long multiwall carbon nanotubes (MWNTs) and the scale bars correspond to 2 mm, 2 mm, and 1 mm respectively. Adapted from ref. 70. Copyright 2011, RSC Advances.

**Fig. 9:**
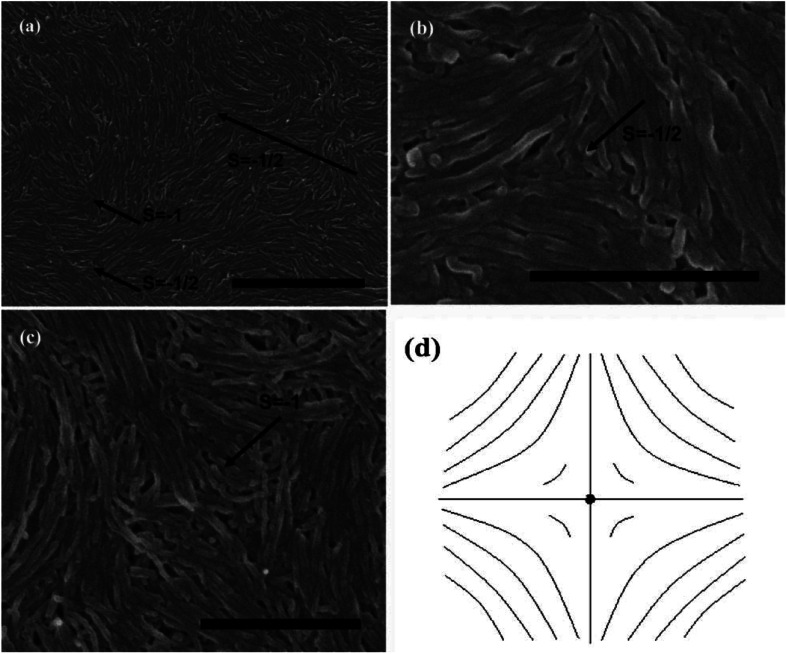
SEM images of the splaying defective cores formed by short MWNTs and the scale bars correspond to 1 mm, 500 nm, and 500 nm respectively. Adapted from ref. 70. Copyright 2011, RSC Advances.

As can be seen in Fig. 7, SEM images clearly show how nanotubes are arranged in the central core of the defects, revealing the orientation properties of nanotubes associated with comparison of *k*_11_ and *k*_33_. From the eqn (1), the energy cost for a certain distortion is proportional to the respective elastic constant, *k*_11_ for splay in the first item and *k*_33_ for bend in the third item. Considering the principle of minimum energy, the lower *k*_*ii*_-related distortion will be energetically favored and most likely to occur. Often one can take into account the analogies between CNTs and LCs to estimate the values of *k*_*ii*_ for nanotubes. For instance, the value of *k*_11_ for polymer LCs is thought to increase as the molecular length and decrease as the molecular diameter, which is convenient to be extended and modified to CNTs. While for *k*_33_, the value of it has been proved to be related to the high Young's modulus *E* and the diameter *D* of nanotubes.^70^ As a result, for short and thick CNTs, the value of *k*_33_ will be calculated larger than that of *k*_11_, and CNTs will have the priority to bend conversely and arrange in the form of splay distortions in negative strength defects (Fig. 8).^70^ If CNTs have larger diameter, the value of *k*_33_ will be calculated much larger than that of *k*_11_; then higher negative strength defects (*i.e. s* = −1) will be observed, where CNTs do not bend and facilitate further the splay deformations (Fig. 9).^70^ However for super-long or ultra-fine CNTs, the value of *k*_11_ may be calculated larger than that of *k*_33_, and instead the positive strength defects will be predominant and stable in turn. Variations in the tube dimensions and rigidity may produce differences between *k*_11_ and *k*_33_. Then comparison of the two material parameters will provide detailed information about the geometry of these defects. As we know it is the term of *k*_11_ ≠ *k*_33_ that denotes the material elastic anisotropy and generates different types of defects. So defects in CNT liquid crystals will successfully reflect the very important material elastic anisotropy associated with *k*_11_ and *k*_33_. But if the value of *k*_11_ is close to that of *k*_33_, all of low energy defects will be observed.^28–30,65,68^

**Fig. 10:**
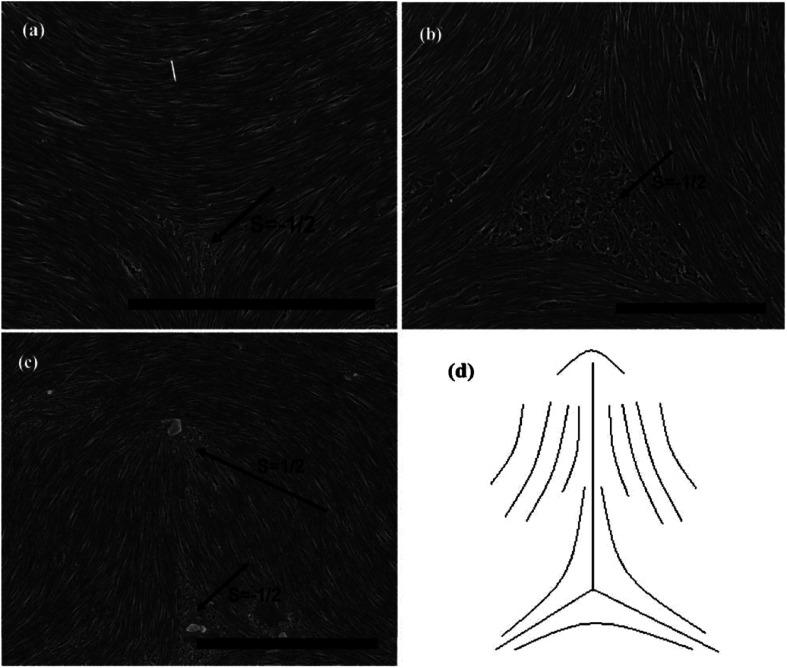
SEM images of the splaying defective cores formed by long single wall carbon nanotubes (SWNTs) and the scale bars correspond to 5 mm, 2 mm, and 5 mm respectively. Adapted from ref. 70. Copyright 2011, RSC Advances.

Subsequently, we consider the interaction between defects associated with splay-bend elastic anisotropy. This resembles the interaction of electrostatic. Defects of like signs repel each another and those of opposite signs attract with the interaction force inversely proportional to the distance. So defects may apt to appear in pairs, *i.e.* neighboring of them connected by brushes should be always of opposite signs. Some examples of the (+1/2, −1/2) pair of defects are shown in Fig. 10(c).^70^ In such pair of defects, the central region still shows the most common splay due to the elastic anisotropy of *k*_11_ < *k*_33_. Here, the presence of +1/2 defects helps the formation of −1/2 splaying cores, *i.e.* the combination of ±1/2 has built the central region rich in splay. It follows that comparison of *k*_11_ and *k*_33_ can also provide the interaction of defects like electrical charges, and the sum of the strengths of all defects in a sample tends to be zero like an electric neutral body as we know it. In particular, the above descriptions do not distinguish single-walled and multi-walled carbon nanotubes. Only because of the lower bending rigidity and the stronger inter-tube van der Waals (vdW) attraction, SWNTs will represent more perfect defective characterization, as is shown in Fig. 10.

In short, if we prepare a nematic sample of CNTs, we will often have defects due to the length and waviness of them. The variation in the tube dimensions and rigidity will produce differences between *k*_11_ and *k*_33_, and lead to different types of defects. Looking at this from another perspective, these defects are still very important in today's research on LCs due to the scanning electron microscope at a resolution sufficient to view individual nanotubes. Here we end the description about liquid-crystalline defects of CNTs by stressing two main aspects. On one hand, CNTs bend differently from rigid rods and do not form random coils like flexible polymers. There is a threshold of the nanotube dimension beyond which a nanotube starts to bend in order to follow the local curvature.^65^ CNTs in the bending state have the high energy barrier for their liquid crystallization by reason of high Young's modulus *E* (the mechanical elasticity), which does not need to address in molecule liquid crystalline systems.^65^ On the other hand, the preferential splaying defects have also been ascribed to the fact that the bend elastic constant *k*_33_ for individual nanotubes are related to the high Young's modulus *E*. Therefore, the value of *k*_33_ is usually calculated greater than that of *k*_11_ for most CNTs with several microns long and several or tens of nanometers or even larger diameter. So the splay distortions will be predominant in such plane sample^70^ and in general these splay defective cores can be mathematically modeled as the similar curves in Fig. 8(d), 9(d) or 10(d), where nanotubes tend to bend and orient themselves conversely inward in order to obtain a differentiable nonzero vector field.

To sum up, CNTs with one-dimensional bending elastic nature can almost reproduce all liquid crystalline defects, and can further serve as a model to examine details of the defective structure due to the large size sufficient to be resolved by the scanning electron microscope. However, such analogy between CNTs and LCs may be somewhat too drastic if real CNT systems are concerned, such as their different scales and different bending elasticity. So either mechanical elasticity or Frank elasticity about nanotubes or the relationships between them are worthwhile to be further studied. Nevertheless, it is the one-dimensional character and the unique elasticity that contribute to the formation of lyotropic liquid crystalline phase, showing obvious physical anisotropy such as electric, magnetic, or optical aspects and showing a certain advantages in the area of display. Then we will introduce the liquid-crystalline application of CNTs in the classic electro-optic device of LCD below.

## 4. Liquid-crystalline applications of CNTs

**Fig. 11:**
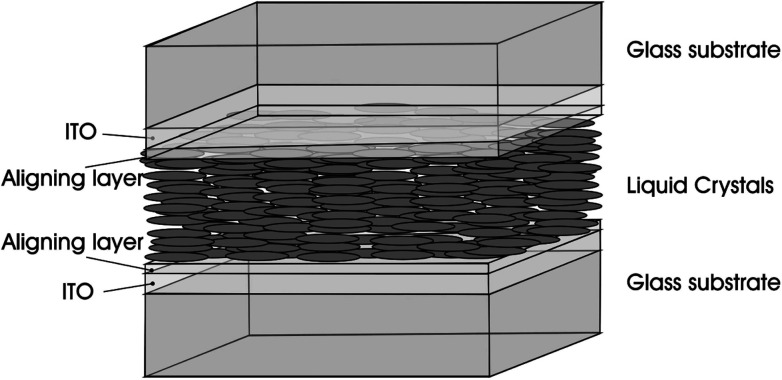
Sketch of a sandwich cell: the LC cell unit. Adapted from ref. 71. Copyright 2010, ChemPhysChem.

It has been known that CNTs have excellent properties when they are in the liquid crystalline state. LCs that are all around us are organic fluids with a degree of order and the typical application is in the area of LCD with the core LC cell unit such as the one in Fig. 11,^71^ composed of two substrates positioned at a desired distance to form an empty gap. In the LC cell unit, transparent electrodes are mostly fabricated from glass panels coated with a thin layer of conducting Indium Tin Oxyde (ITO), and the Polyimide (PI) aligning layer can be fabricated by means of the rubbing process. As for LCs, they are located in the middle layer and can be anchored on the substrate in different forms to prepare LCD devices with different modes.

Nowadays, LCD market has greatly extended and developed from small-size mobile phones to large-size LCD-television (LCD-TV). However, continuous improvements in the performance of electro-optic display are necessary to meet the customer's requirements such as wide viewing angle, fast response time, high resolution, low power consumption, high color reproducibility, and *etc.* With this basic understanding in place, liquid crystalline CNTs may work complementarily and play potential roles in the field of LCD as guests in LC layers, as electrodes, as alignment layers or as the polarizers in the LC cell unit. Below we will introduce these aspects respectively.

### 4.1 CNTs as guests in LC layers

Because of the small dimensions, a small amount of CNTs will not create distortions of the LC matrix. Dispersing CNTs in LCs, CNTs will align along the LCs' director and share their intrinsic properties with the LC matrix,^52,72–77^*e.g.* the enhanced dielectric properties of the LC-CNT dispersion. On one hand, LC molecules will interact strongly with the CNT surface *via* the π–π stacking. This kind of interaction between CNTs and LCs is strong with a binding energy of about −2 eV for the length of CNTs is on average much longer than that of LCs.^43,72,76^ On the other hand, there is also elastic interaction between CNTs and LCs *via* the director field, which will further promote CNTs parallel to LCs' director.^34,36,49,72,77^ Since the elastic modulus of most CNTs is often estimated to be much larger than that of LCs, the elastic energy for composite of them will increase due to the addition of nanotubes.^78^ In addition, when introducing CNTs into LCs, CNTs may also provide an effective electron exchange due to their large electron affinity.^36,40,41^ A richer discussion about this issue will be seen in the following chapters.

**Fig. 12:**
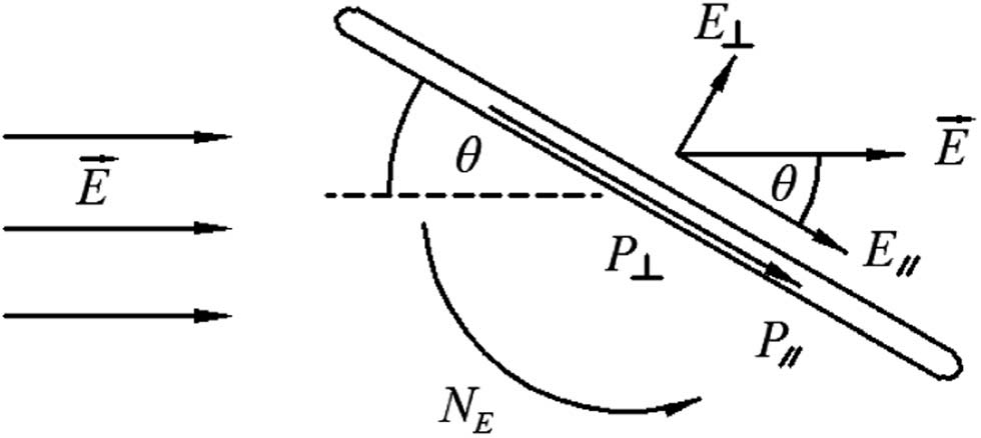
An illustration of the behavior of a cylindrical particle exposed to a homogeneous electric field. Adapted from ref. 79. Copyright 2005, Polymer.

As we know both of electric and magnetic fields can orient CNTs in liquid crystalline state. Here we introduce the dynamic orientation of CNTs in the presence of an electric field. By applying the electric field *E⃑*, each conductive nanotube will experience the polarization, and the induced dipole moment *P⃑* will be proportional to the field strength and the permittivity.^36^ An illustration of the behavior of the equivalent model of cylindrical particle exposed to a homogeneous electric field is given in Fig. 12.^79^ As shown, *P⃑* can be divided into 
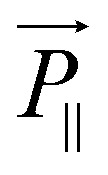
 and 
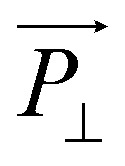
, and 
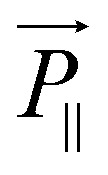
 will be much larger than 
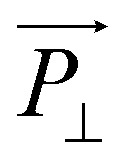
 because of the positive dielectric anisotropy of a nanotube. Then this polarization will produce the torque *N*_*E*_ to align the nanotube in the direction of the electric field and minimize the dipolar energy. In addition, this polarization may also lead to an additional attractive interaction between ends of the dispersed nanotubes, producing a conductive nanotube network.^79–81^ So CNTs are expected to induce distinctive changes to the physical properties of the LC matrix and further bring the enhanced performances of LCs for display.

**Fig. 13:**
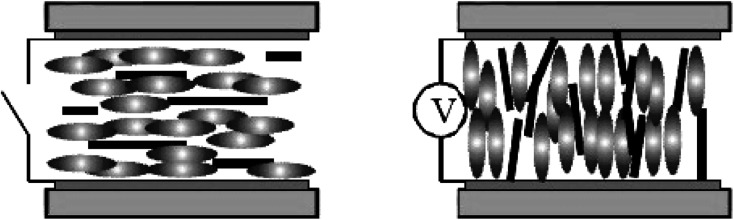
Sketch of mixtures of carbon nanotubes and liquid crystals inserted in a standard LC cell.

In LCD, LCs can successfully orient CNTs dispersed in them (Fig. 13) and with the effects of nanotubes the physical properties of the LC matrix will be greatly improved.^34,36,37,41,82–91^ When an external electric field is applied, LC molecules with positive dielectric anisotropy will tend to align along the field and then prompt CNTs to reorient accordingly *via* the director field or the dipolar interaction. As a result, the doped nanotubes will influence the elastic constant and dielectric properties of the LC-CNT dispersion by the experiment.^91^ For the former, both of the twist (*k*_22_) and bend (*k*_33_) elastic constant will exhibit an increase,^35,45,78^ while the splay (*k*_11_) elastic constant will show increase^78^ or decrease^78,91^ depending on the orientational order of the mixture, and for the latter, the dielectric properties will also be enhanced due to the alignment of nanotubes in LCs in LCD unit.^91^ In addition, the enhanced cell dielectric constants are also attributed to the charge exchange between the cell electrode and ions presented in LCs by means of the dispersed nanotubes.^36,37^ Initially, the strong interaction between CNTs and LCs will make the electrostatic charge transfer from the LC molecule to CNTs. Once CNTs become in contact with the electrodes, the charge will then transfer from CNTs to electrodes. As a result, by participation of nanotubes the cell conductivity will increase. And the final population of adsorbed ion charges in the alignment layer will increase and cause the screening effect that will decrease the effect voltage of the LC medium. Although CNTs may further decrease the effect voltage due to the electric field enhanced ordering resulted from the fact that CNTs often have agglomerating tendency and have not perfect straight form while dispersed in an LC, they will have sufficient ion trapping ability to rectify the interface properties and make the double-layer effect less effective to decrease the observed amount of moving charges. So the screening effect can be effectively reduced. Besides, nanotubes insertion in an LC might significantly reduce the system viscosity^41,45^ or increase the clear-point of the composite.^51^ Also, the incorporated CNTs might play a vital role in improving the thermal stability of the system or widen the working temperature range in liquid crystalline phase.^37,83,87^

Next, notable performance benefits of introducing CNTs into the LC matrix in LCD will be illustrated below. Firstly, the threshold voltage will be reduced with the increase of the concentration of CNTs for several reasons.^33,37,40,41,90–96^ One of them is the enhanced dielectric anisotropy of the LC-CNT dispersion. Another is the reduced screen effect caused by reduced ion charges within the cells. And the third might be the electric-field enhancement effect from CNTs that will induce a very strong localized electric field due to the high aspect ratio. Secondly, the response time will be improved for reasons as follows: the suppression of the field screening effect, the increase in the dielectric anisotropy, the increase of the elastic constant as well as the decrease in the viscosity.^35,45,97–101^ For example, a faster response time can be achieved in a patterned vertical aligned (PVA) LC cell due to the increased bend elastic constant,^35^ and in an in-plane switching LC cell, a faster response time can also be achieved due to the increased twist elastic constant.^45^ Thirdly, the insertion of CNTs into LCs may also enhance or maintain the transparency of LC cells that is a useful indicator for display quality.^37^ The CNT-doped LC cells will have very high transmittance characteristics compared with pure LC cells under the prerequisite of the well-dispersed and well-reoriented nanotubes in the LC matrix.

So to achieve above notable benefits CNTs should be well dispersed and aligned in the surrounding LC matrix. Although dispersing CNTs in fluids at sufficient concentration is a considerable challenge, there have been many approaches to overcome this obstacle and CNTs have been successfully dispersed in thermotropic or lyotropic liquid crystals.^102–104^ Maybe we can improve the compatibility between CNTs and the matrix by decorating the CNT surface with the matter being structurally similar to that of the matrix, as in ref. 104. It is expected that the well dispersion and alignment of CNTs in LCs would pave a promising way toward the future relevant academic and industrial research.

### 4.2 CNT films as electrodes

**Fig. 14:**
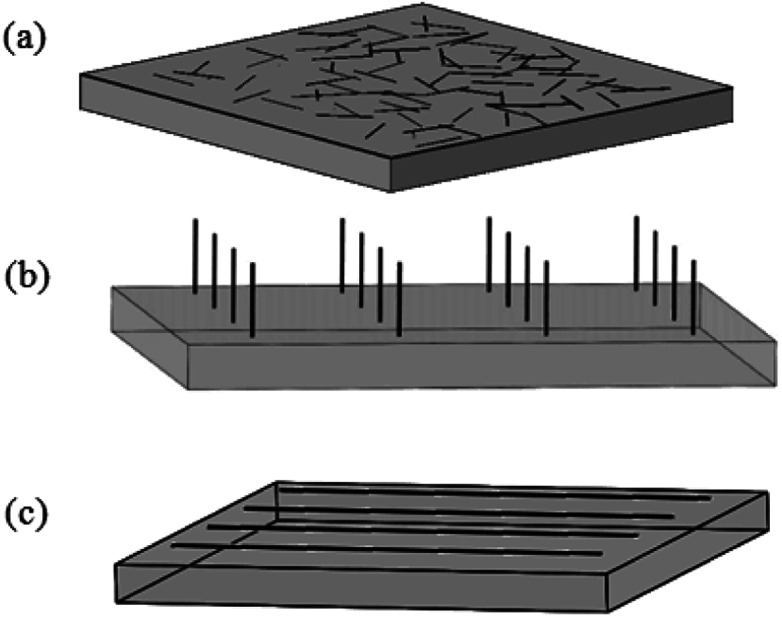
Sketch of CNT films in the form of (a) CNT network, (b) vertically-aligned-CNT array or (c) horizontally-aligned-CNT arrays.

It is well-known that CNTs are very good electric conductors and can be likened to conductive metal rods of nanometer dimensions. The electrical conductivity in the tube radial direction is much lower than that in the axis direction. This is because that when electrons travel perpendicular to the tube axis, they need to hop from one graphitic layer to another causing a lower electrical conductivity in the radial direction. Then for CNT arrays, the electrical conductivity will be about an order of magnitude higher along the tube axis. So CNT films with the structure of CNT network, vertically-aligned-CNT array or horizontally-aligned-CNT arrays (Fig. 14) will have considerable potential to be used as the efficient nano-structured electrodes due to the axial conductivity.

Here we introduce such conductive films of CNTs as electrodes in the field of LCD. In the LC cell unit, conventional ITO electrodes have a number of disadvantages and will not be the excellent one in future optoelectronic devices. The difficulties of ITO revolve around the rising cost of indium, the brittleness of ITO, as well as the high temperature and vacuum processing used in the production.^31,32,44^ While CNT films have advantages in low cost due to the natural abundance of carbon, flexible or solution processed application, amenability to spray and printing process.^31,32,44,105^ So they are hopeful to overcome the above mentioned disadvantages and to be the substitute for ITO.

In one case, both of LCs' orientation behavior and the electro-optic characteristics exhibit the suitability of CNT network electrodes (Fig. 14(a)) for practical application in LCD. Taking a twisted alignment mode for an example, the electro-optic characteristics have been effectively improved and the lowered switching amplitude has also been observed due to the electric-field enhancement effect resulted from CNTs with a high aspect ratio.^44^ Also due to the randomly organized nanotubes, interesting director profile arrangements within the LC layers may be produced and can be electrically controlled, which may open opportunities for a wide range of applications in the devices, such as optical diffusers, beam shaping and random laser.^106^

In another case, oriented CNTs (vertically- or horizontally-aligned-CNT arrays) electrodes (Fig. 14(b) and (c)) will also exhibit excellent optical transmission and electrical conduction. For vertically-aligned-CNT array, the vertical CNTs in arrays will act as individual electrode sites which spawn more electric field due to the large aspect ratio, forming the electrode array. Once the concentrated electric field around the nanotubes is exposed to an alternating voltage, it will become strong enough to polarize the LC molecules at a low voltage. Then LC molecules with positive dielectric anisotropy will align parallel to the electric field and exhibit the freedom to flow.^107^ So the electrodes of vertical aligned nanotubes are expected to optimize the device's performances, such as the voltage-transmission (*V*–*T*) characteristic, response time and the contrast ratio.^108^ And so do horizontally-aligned-CNT arrays. The horizontal CNT arrays will exhibit significant electrical anisotropy and the low sheet resistance.^109,110^ Such CNT arrays can show good modulation of the transmittance by the electric field and less residual direct current (DC) property.^110^ In addition, the electrodes of horizontal aligned nanotubes will further reduce the path lengths and improve the carrier mobility.^32,111,112^ Also they can be used as the heating electrode which will extend the working temperature range of the LCDs.^31^

Overall, all of these promising results demonstrate that CNT films will have great application potential in the field of LCD as the electrodes. They will become a promising candidate to substitute the current ITO electrodes for the future research related to display or other electro-optic devices.

### 4.3 CNT films as alignment layers

Along with the electrodes, alignment layers are also an important part for next generation of LCD. CNT arrays can align LC molecules *via* the director fields due to the common one-dimensional structure and the π–π interactions between them. Although both of the anchoring energy and the dipole–dipole interaction are relatively weak, the pretilt angle can still be produced and the threshold voltage can also be lowered in the adaptable LC modes.^32^ Moreover, considering the aligning mechanism in LCD, the equivalent grooves to the traditional PI are shown in the Fig. 14(c).^31^ The drawing direction is equivalent to the rubbing direction of PI, which will play a vital role in aligning LC molecules. Plus the film is easy to scale up to a large area and can be fabricated into a precise micro pattern by laser cutting or lithography for the convenience of commercial exploitation. So the films of aligned CNTs are hopeful to replace the traditional PI and prompt LCs' alignment in LCD.

**Fig. 15:**
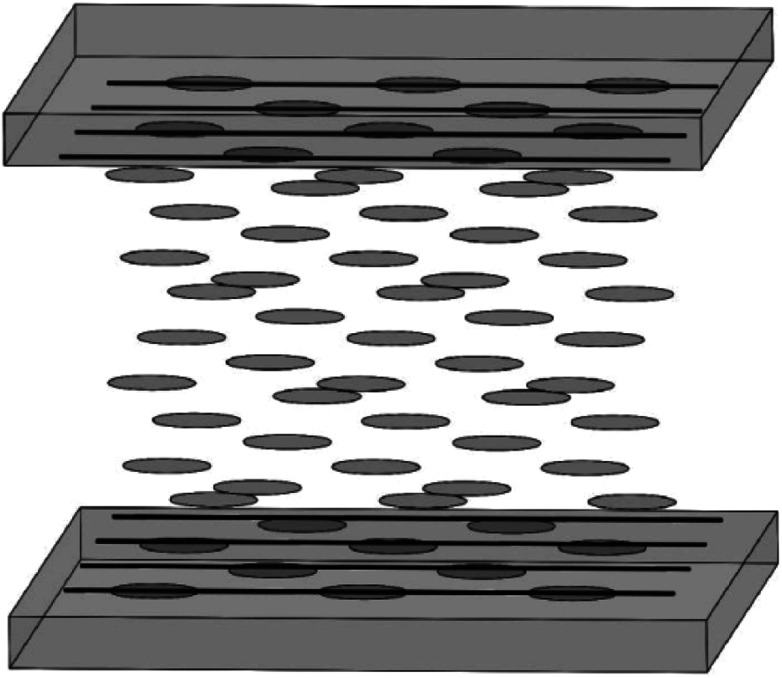
Sketch of the aligning of LC molecules by CNT films. The upper panel and the lower panel have the same structure and were placed parallel to each other.

**Fig. 16:**
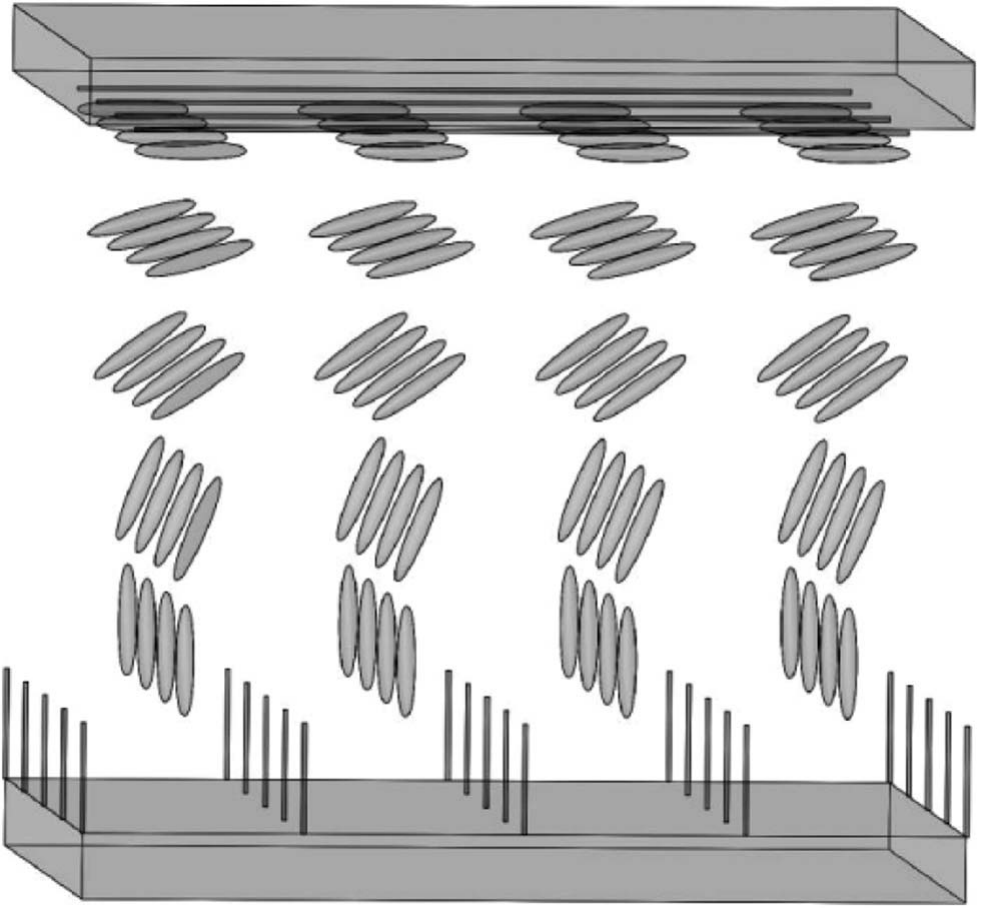
Sketch of the aligning of LC molecules by CNT films. The upper panel is the horizontally-aligned-CNT array and the lower panel is the vertically-aligned-CNT array.

Fig. 15 is a schematic illustration of the LCD cell based upon a horizontally-aligned-CNT array film, where CNT arrays are used as the alignment layers for LC molecules. Both of the upper alignment layers and the lower ones are composed of a plurality of parallel and spaced CNTs. The nano-grooves in the film will determine the aligning of LC molecules. As can be seen in the figure, a homogeneous alignment of LC molecules will be obtained from the randomly oriented nematic phase (a Schlieren texture).^113^ Then if the bottom layer is the vertical CNT array, the configuration of the device will be the hybrid alignment mode (Fig. 16), where LC molecules in the cell will have a hybrid distribution. And because CNT arrays are also efficient nanostructured electrodes as described above, they will be a good substitute for both of ITO and PI. Taking pentylcyanobiphenyl (5CB) molecules as an example, upon application of an electric field, the nematic director will rotate from planar to homeotropic due to the positive dielectric anisotropy (similar to that in Fig. 13), showing CNT arrays can successfully control the orientation of LC molecules and at the same time also act as a conductive layer.^32,114^ This can also be seen from the literature,^110^ where CNT arrays can function both as the alignment layer and electrode, thus reducing the production cost.

Overall, oriented CNTs can be used to align LC molecules as the alignment layers and can exhibit a low sheet resistance as the nanostructured electrodes. The films of oriented CNTs will become both of aligning and conducting coatings for next generations of LCD devices.

Besides, it should be noted that films of a mixture of CNT and PI working as the coatings on an ITO glass substrate have also been investigated.^115–117^ Y. Liu *et al.* have made the composite film in a twisted nematic LCD.^115^ In this investigation, the authors demonstrated an overall improvement of around 16.12% in the total switching time and explained this effect by considering the electric field enhancement effect caused by the CNT clusters, the strong anchoring strength between LCs and CNT clusters, the decrease in the effective cell gap, as well as the bumps on the alignment layer's surface. And in the work by H. Lee *et al.*, the authors have also reported the improvement of the relaxation time and the order parameter of nematic LCs using such films mixed with CNTs and PI.^116^ Hence, the CNT-PI mixtures are also expected to be very useful for practical applications of LCDs.

### 4.4 CNT films as polarizers

**Fig. 17:**
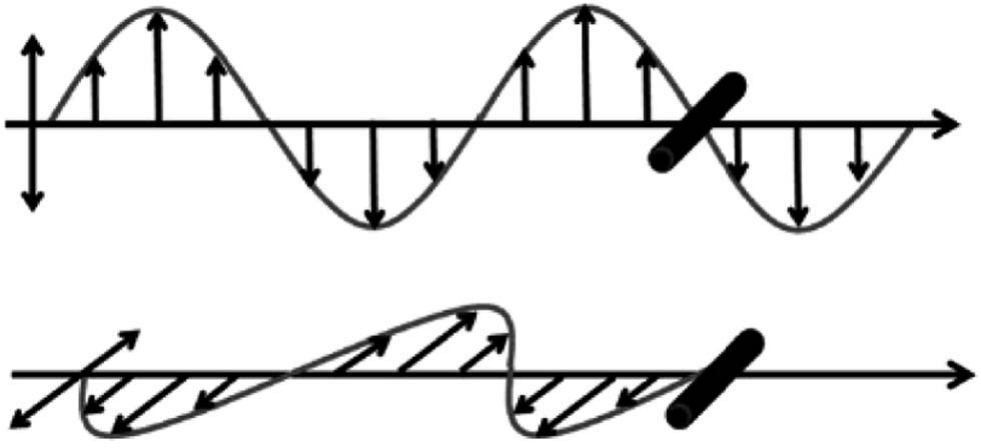
Schematic diagram showing light absorption of incident light with *E*_‖_ and *E*_⊥_ by CNTs.

**Fig. 18:**
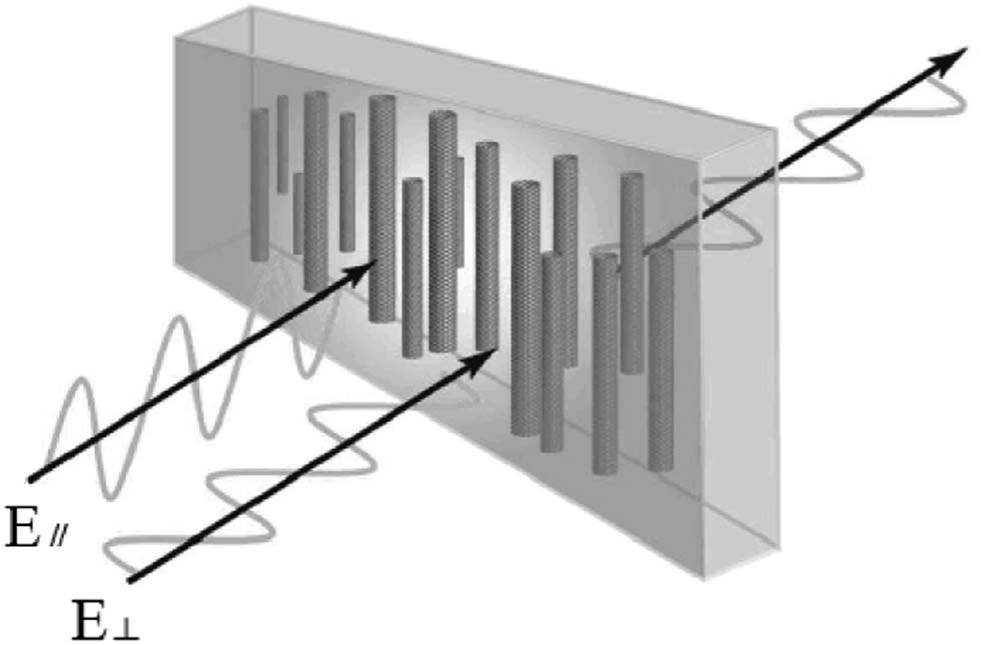
Sketch of the aligned CNT polarizer.

Due to the one-dimensional anisotropy and the graphite π electrons, CNTs exhibit anisotropic optical responses similar to a dichroic dye.^118,119^ There will be no attenuation or strong absorption when the light polarization is perpendicular (*E*_⊥_) or parallel (*E*_‖_) to the nanotube axis, as shown in Fig. 17. Then by means of an external magnetic or electric or other field, a strongly absorbing film of highly aligned CNTs can be obtained as illustrated in the schematic of Fig. 18.^120^ And such anisotropic absorption will exist in the visible region, as well as in ultraviolet and infrared regions, which will be attractive for practical optical applications.^119^

**Fig. 19:**
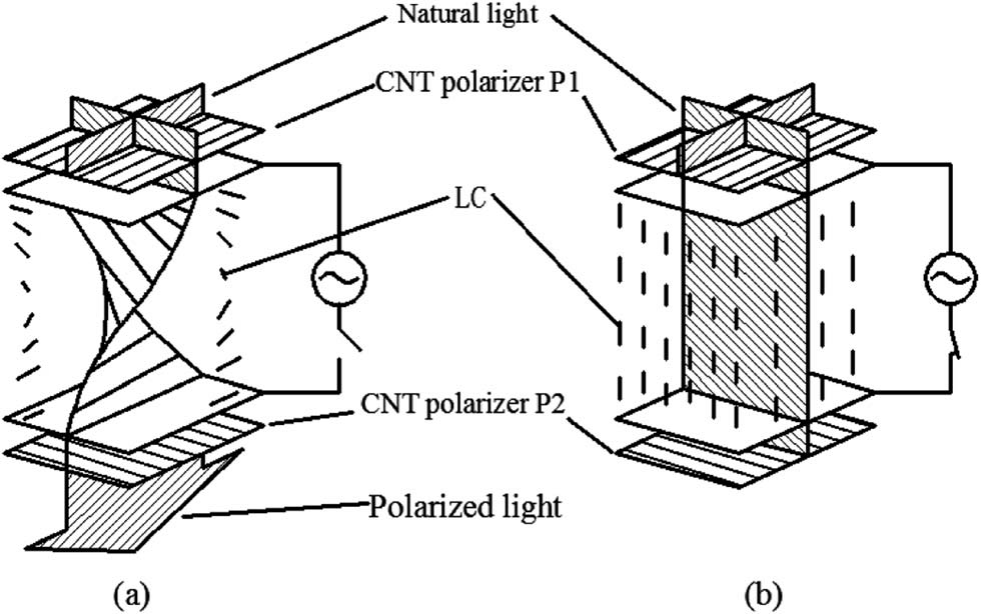
Schematic cell structures of a TN-LCD by the aligned CNT polarizer.

For example, we can fabricate the optical polarizer based upon the film.^121^ The stretched CNTs (or the clusters) in the film will absorb or transmit incident light with its electric vector propagating parallel or perpendicular to the long axis. So the degree of alignment of them in the film will demonstrate that of the anisotropic optical absorbance. Then such optical polarizer can be applied in the field of LCD in Fig. 19. Without a bias field, the natural light will pass through the CNT polarizer P1, leaving only linearly polarized light perpendicular to the long axis of nanotubes to inject into LC layers. Because the polarization direction of the linearly polarized light is parallel to the molecular orientation (taking positive LCs as an example) at the incident plane, the polarization direction will rotate synchronously with the LC molecular in TN-LCDs. So the polarized light will penetrate the cell for crossed CNTs polarizers as shown in Fig. 19(a) and the cell will show a white state. When vertical electric field is applied as shown in Fig. 19(b), LC molecules will be parallel to the electric field due to the positive dielectric anisotropy and the polarization direction of the polarized light will maintain its original direction to the output plane. Then the polarizing axis will be parallel to the long axis of nanotubes so the cell will appear to be black. Furthermore the film will exhibit the degree of polarization (DOP) of 90% due to the strong anisotropy of nanotubes and keep almost constant through the wide spectral range due to the broad absorption spectrum from ultraviolet to near infrared.

Besides, thanks to the controlled and modulated selective light absorption by different sizes of stretched CNTs (or the clusters) under external fields, CNT polarizers will possibly be applied to dynamic polarizers and light modulators by controlling the polarization and light modulation of visible light. As well as, thanks to the chemical and thermal robustness of nanotubes, CNT-based optical polarizer would have a potential to be used for intense light or under severe thermal or chemical circumstances in spite of a relative low transmittance compared to the ordinary polarizer.

## 5. Conclusions and outlook

In the final part, we give a summary and outlook. Similar to conventional LCs, CNTs have liquid crystallinity and can almost present all kinds of liquid crystalline defects. CNTs are expected to exist in the liquid crystalline state based upon the competition of translational and rotational entropy from the well-established Onsager's theory provided the control factors are properly regulated, such as the solubility and dispersibility, purification and the interaction between tubes. The exploration of Frank elasticity to nanotubes and the resultant defects will contribute to understand the formation of liquid crystalline phase as well as the relevant applications, especially in the area of display. However, little has been done in this direction. In this paper we have reviewed the basic structural properties of CNT liquid crystalline defects and the relevant electronic application of CNTs in LCD. There is still much more to explore if one considers the effect of waviness or deformation of real CNTs, the length polydispersity, as well as the field-effect behavior. Although most of them are still at a relatively early explorative stage, we hope this brief review will inspire the readers as much as the authors are inspired by the research discussed. These current limitations and challenges are bound to lay down the foundation for future developments or to provide a promising route for further explorations. CNT liquid crystals are worthwhile to be further studied and will certainly develop into a highly successful research field after a few years, bringing a bright and rich future for the practical applications in various forms of CNT-based devices especially in LCD.

## Conflicts of interest

There are no conflicts to declare.

## Supplementary Material
